# Clinical outcomes of an intelligent pressure-controlled disposable ureteroscope for Laser lithotripsy in renal stone surgery: a retrospective matched cohort study

**DOI:** 10.3389/fsurg.2025.1637385

**Published:** 2025-10-28

**Authors:** Zhong Lv, Zhimin Jiao, Honglei Shi, Xiaoliang Yuan, Tingchun Wu, Haoran Wu

**Affiliations:** 1 Department of Urology, Wujin Hospital Affiliated with Jiangsu University, Changzhou, Jiangsu, China; 2 The Wujin Clinical College of Xuzhou Medical University, Changzhou, Jiangsu, China

**Keywords:** upper, urinary, tract, calculi, intelligent, pressure-control, ureteroscope, inflammation

## Abstract

**Purpose:**

To compare the clinical application efficacy of an intelligent pressure-controlled disposable ureteroscope vs. a conventional disposable ureteroscope with laser lithotripsy in upper urinary tract calculi surgeries.

**Methods:**

The experimental group (*n* = 70) underwent surgery using an intelligent pressure-controlled disposable ureteroscope with laser lithotripsy, while the control group (*n* = 70) underwent traditional disposable ureteroscope Holmium laser lithotripsy. The perioperative conditions of patients in both groups were observed, including average surgical time, postoperative hemoglobin loss, average hospital stay, renal pelvis pressure, blood urea nitrogen (BUN), serum creatinine (Scr), platelets (PLT), infection and inflammation indicators, initial stone clearance rate, and total stone clearance rate. The occurrence rate of postoperative complications in both groups was assessed using the Clavien-Dindo classification, and the differences in complication rates between the groups were compared.

**Results:**

The experimental group had a longer surgical time, but a shorter hospital stay and lower renal pelvis pressure. Inflammatory markers including WBC, CRP, and PCT were significantly lower postoperatively in the experimental group. Initial stone clearance and total clearance rates were also higher. The overall complication rate was significantly lower in the experimental group

**Conclusions:**

The use of an intelligent pressure-controlled disposable ureteroscope with laser lithotripsy for upper urinary tract surgeries can effectively reduce postoperative inflammation indicators, decrease average hospital stay and renal pelvis pressure, and improve the initial and total stone clearance rates.

## Introduction

Upper urinary tract calculi represent a significant global public health concern (1, 2), with various clinical treatment modalities continuously being evaluated to identify the optimal therapeutic strategy. Traditional ureteroscopic lithotripsy is widely employed in the management of upper urinary tract stones (3). However, potential issues such as elevated renal pelvic pressure that can lead to urinary tract infections, perirenal extravasation, and potential renal function impairment, as well as postoperative hemoglobin loss, and heightened inflammatory responses associated with this surgical method have presented considerable challenges to both treatment outcomes and patient recovery (4–6). To address these clinical concerns, we conducted a comparative study evaluating the intelligent pressure-controlled disposable ureteroscope vs. a conventional disposable ureteroscope in terms of efficacy and safety. The emergence of intelligent pressure-controlled disposable ureteroscope with laser lithotripsy in recent years has offered potential solutions to these issues (7). By incorporating intelligent pressure control technology, utilizing negative pressure aspiration, renal pelvic pressure monitoring, and high-flow perfusion, this technique aims to reduce the temperature during Holmium laser lithotripsy, theoretically lowering renal pelvic pressure, enhancing stone clearance rates, and thereby minimizing the incidence of postoperative complications. Although the intelligent pressure-controlled disposable ureteroscope with laser lithotripsy has demonstrated potential in addressing these complications, further research is required to validate its effectiveness and safety in clinical practice (8). Thus, we conducted this study to evaluate the actual performance and safety of the intelligent pressure-controlled disposable ureteroscope with laser lithotripsy in upper urinary tract stone surgeries, aiming to provide robust evidence for its clinical application in managing upper urinary tract calculi.

## Methods

### Study design

This study was a retrospective cohort study. Medical records of 140 patients who underwent surgery for upper urinary tract calculi at the Department of Urology, Wujin People's Hospital between May 2021 and May 2023 were reviewed. Although patients were not randomized and the surgical method was chosen based on surgeon preference and clinical availability, we subsequently applied a matched cohort selection strategy to minimize confounding and selection bias. Specifically, we included an equal number of patients (*n* = 70) in each group by matching based on treatment period, surgical indication, and completeness of clinical records.

This matched cohort design has been widely applied in retrospective studies to improve comparability between treatment groups and enhance the robustness of statistical comparisons (8).

### General information

A cohort of 140 patients undergoing surgery for upper urinary tract calculi at the Department of Urology, Wujin People's Hospital, Changzhou, Jiangsu Province, from May 2021 to May 2023, was selected as the study population. These individuals were allocated into two groups based on the surgical technique utilized, with 70 patients in each group. The equal sample sizes between groups were the result of intentional matching within the retrospective data to improve baseline comparability. Prior to the treatment, all patients underwent a comprehensive medical evaluation, which included history taking, physical examination, laboratory investigations (such as blood tests and urine culture to rule out infection), and radiological assessments. All patients also received standardized perioperative antibiotic prophylaxis. Specifically, a single dose of intravenous cefuroxime (1.5 g) was administered 30–60 min prior to surgery. In patients with a confirmed positive urine culture or heightened risk of infection, broader-spectrum antibiotics such as piperacillin-tazobactam or levofloxacin were administered based on sensitivity testing. Surgical procedures were scheduled only after clinical improvement was confirmed—such as resolution of fever and normalization of inflammatory markers—ensuring the infection was under control in accordance with our institutional infection management protocols.

In patients with obstructive symptoms or clinical signs suggestive of progressive infection, a staged approach was adopted in accordance with clinical guidelines. Specifically, initial management included ureteral stenting or percutaneous nephrostomy for urinary drainage combined with targeted antibiotic therapy until infection control was achieved. Definitive stone surgery was scheduled electively once inflammatory markers normalized and the infection was resolved, except in rare cases requiring urgent intervention due to rapidly deteriorating clinical status. These cases were performed under stringent perioperative monitoring.

**Figure 1:**
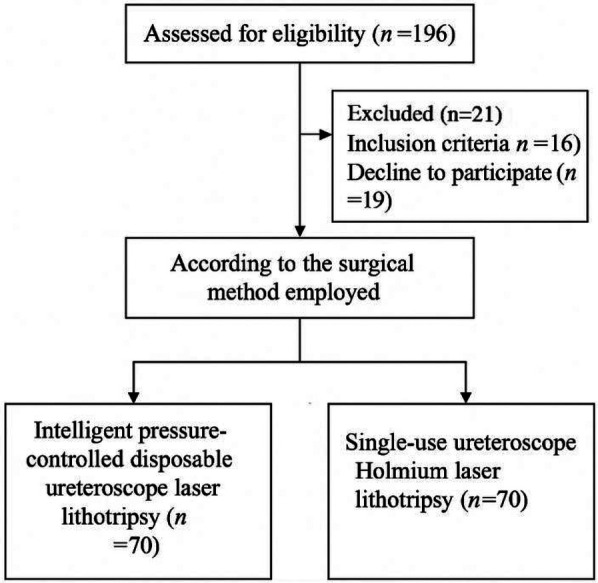
CONSORT flow diagram showing patient enrollment, group allocation, and analysis.

Baseline characteristics of the patients in both groups are summarized in Table 1. The patient recruitment, allocation, and analysis process is illustrated in the CONSORT flow diagram (Figure 1).

**Table 1:** Comparison of baseline characteristics between the Two groups.

Characteristic	Experimental group (*n* = 70)	Control group (*n* = 70)	*t*/*X*^2^	*P*-value
Gender				
Male (*n*%)	58	55	0.458	0.857
Female (*n*%)	12	15		
Age (years, $\bar{x}\pm s$)	51.56 ± 6.53	53.49 ± 10.13	0.427	0.513
BMI (kg/m^2^, $\bar{x}\pm s$)	22.64 ± 3.52	23.14 ± 3.46	0.524	0.627
Maximum stone diameter (mm, $\bar{x}\pm s$)	20.87 ± 3.08	21.24 ± 2.54	0.647	0.425
Stone location			0.627	0.582
Right (*n*%)	32	41		
Left (*n*%)	38	29		
Hydronephrosis				
None (*n*%)	3	5	0.651	0.574
Mild (*n*%)	56	51		
Moderate or above (*n*%)	11	14		
Staghorn calculus morphology				
Incomplete type (*n*%)	2	3	0.887	0.925
Complete type (*n*%)	0	0		
Stone CT value (Hounsfield unit, Hu, $\bar{x}\pm s$)	728.78 ± 89.25	743.01 ± 101.18	0.412	0.617

“*t*/*χ*^2^” indicates the type of statistical test applied: *t*-values are used for continuous variables (independent samples *t*-test), and *χ*^2^-values are used for categorical variables (Chi-square test). *P*-values <0.05 were considered statistically significant.

### Inclusion and exclusion criteria

Inclusion Criteria: Patients diagnosed with upper urinary tract calculi via abdominal CT, KUB, or CT urography; classified as American Society of Anesthesiologists (ASA) grade I–II; indications for surgery present. Stone size greater than 0.5 cm and less than 3 cm; all enrolled patients routinely received ipsilateral ureteral D-J (double-J) stent placement 1–2 weeks prior to surgery as part of standardized preoperative ureteral dilation, ensuring optimal access and minimizing the risk of ureteral injury during the procedure; All enrolled participants were legally competent to provide informed consent. Patients or their family members agreed to participate in the study and signed an informed consent form. For patients with limited decision-making capacity, informed consent was obtained from legally authorized representatives.

Exclusion Criteria: Patients with severe cardiopulmonary diseases, significant renal impairment, or hemorrhagic disorders; contraindications to surgery; pregnant women, infants, morbidly obese individuals, or patients with spinal deformities; renal or ureteral anomalies such as duplicated kidney, ectopic kidney, horseshoe kidney, or ureteropelvic junction obstruction; uncontrolled urinary tract infection (defined as symptomatic infection with systemic signs such as fever >38.5 °C, flank pain, chills, and a positive urine culture, unresponsive to appropriate antibiotic therapy within 72 h); patients with concomitant tumors; pregnant or lactating women.

### Surgical methods

All surgeries were conducted by a fixed surgical team, including a lead surgeon, a first assistant, and a second assistant. The same team performed all procedures during the study period. This approach was designed to minimize complications and prevent instrument damage due to operator unfamiliarity, thereby reducing patient costs and the burden on medical insurance. The lead surgeon performed all critical steps of the procedures to ensure technical consistency.

### Control group procedure

For the control group, we performed traditional single-use ureteroscope Holmium laser lithotripsy (Zhuhai Pusen Medical Technology Co., Ltd., Registration Certificate Number: Yue Machinery Approval No. 20212060834) as follows: Under general anesthesia, the patient was positioned in the lithotomy position. 1. We used an F8/9.8 WOLF rigid ureteroscope to remove the pre-placed D-J stent, and We employed a COOK malleable guidewire under direct endoscopic visualization to access the renal pelvis accurately, ensuring the correct placement of subsequent instruments. The scope was then retracted, and a standard 12/14F COOK ureteral access sheath (length 35 cm) was inserted as the working channel. 2. An F7.5 PUSEN disposable ureteroscope was advanced into the renal pelvis, with manual saline infusion using the Olympus UHI-4 irrigation pump by the assistant to locate the stone. 3. We performed Holmium laser lithotripsy by using 220/235 um laser fiber settings at 0.8–1.2 J energy and 10–20 Hz frequency; during lithotripsy, the laser's energy and frequency were controlled to minimize damage to surrounding tissues. Larger stones were typically managed with a stone basket or foreign body forceps for enhanced removal. 4. Before concluding the surgery, We placed a 6F D-J stent in the ureteropelvic junction, left *in situ* for four weeks. In order to monitor intrarenal pressure in the control group, a 0.014-inch PressureWire (St. Jude Medical, USA) was inserted into the renal cavities during the procedure. Real-time measurements of intrapelvic pressure (IPP) were recorded during flexible ureterorenoscopy, based on the method described by Sierra et al. (9).

### Experimental group procedure

**Figure 2:**
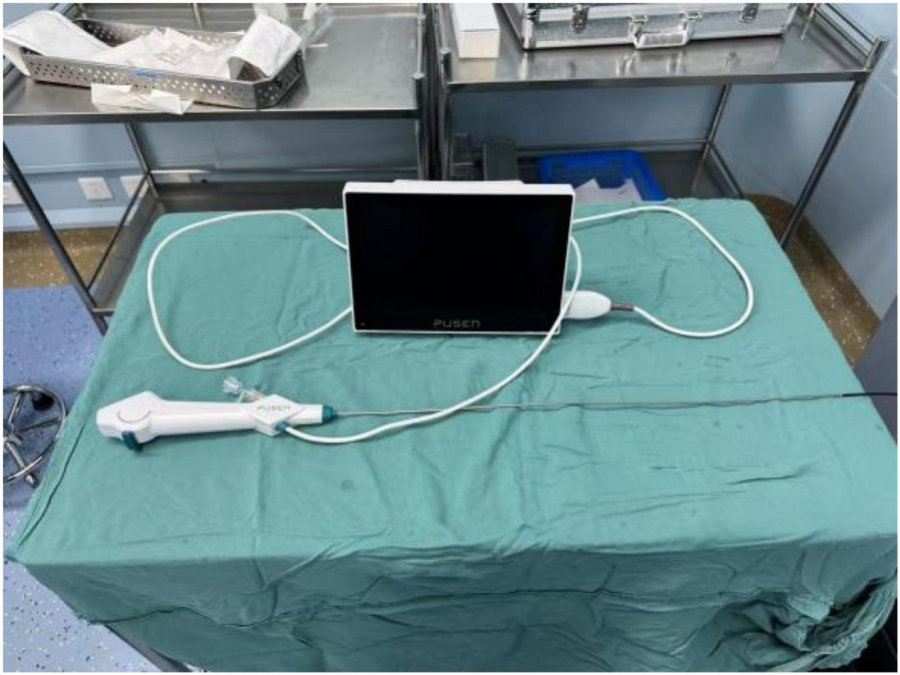
Traditional single-use ureteroscope with Holmium laser lithotripsy system used in the control group. (Zhuhai Pusen Medical Technology Co., Ltd. Registration Certificate Number: Yue Machinery Approval No. 20212060834).

**Figure 3:**
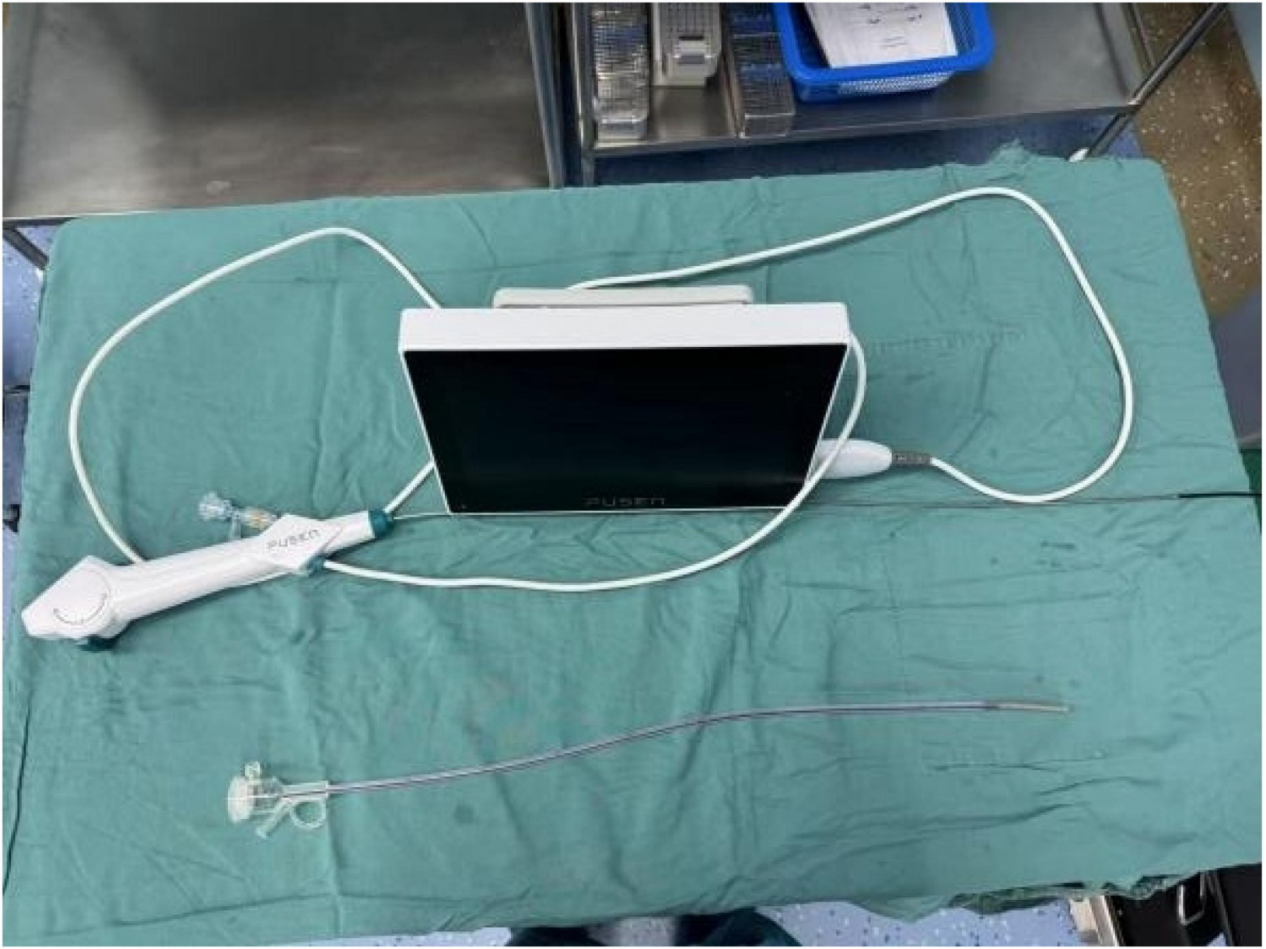
Medivators pressure-sensing aspiration ureteral sheath used in the experimental group. (Jiangxi Yweit Special Technology Co., Ltd. Registration Certificate Number: Gan Machinery Approval No. 20192060357).

**Figure 4:**
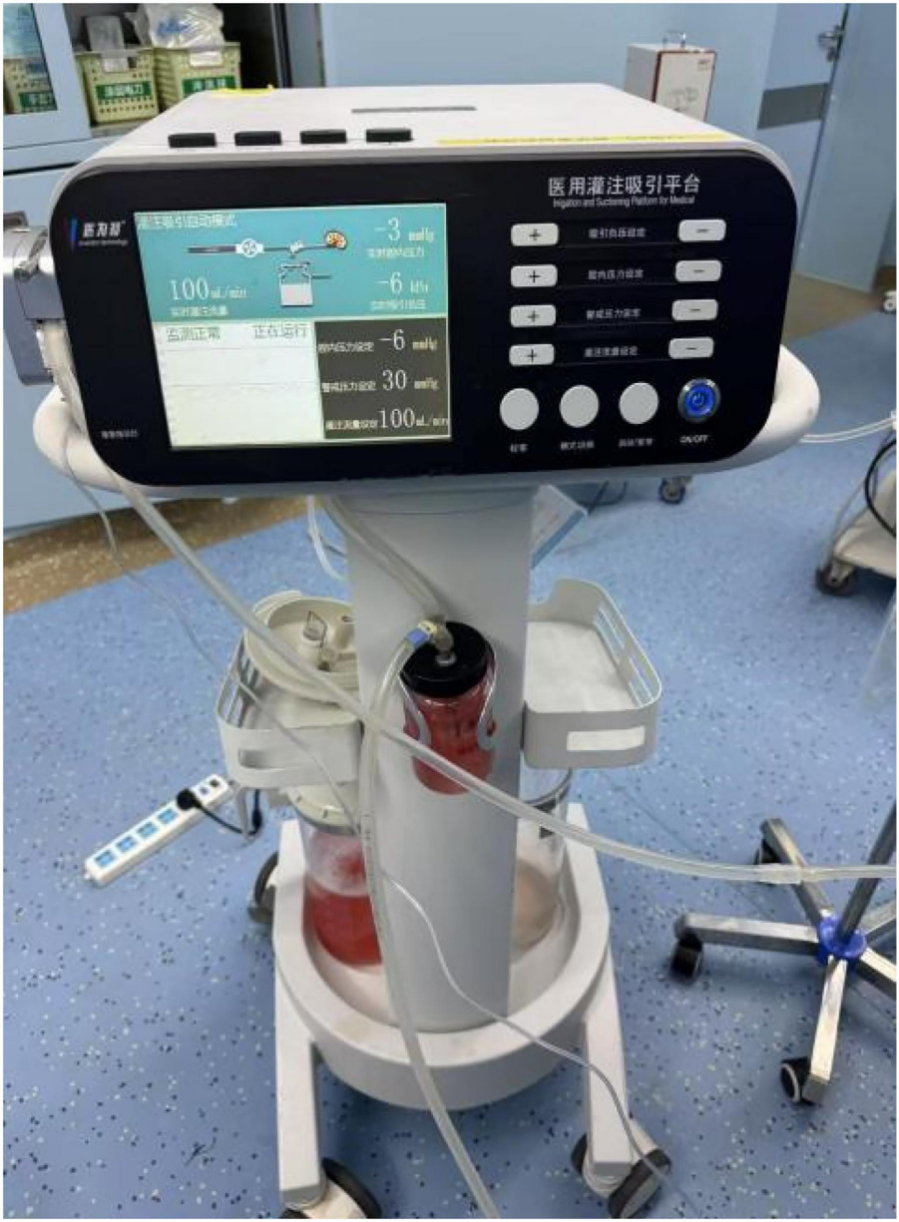
Infusion and aspiration platform with the screen showing real-time intracavitary pressure and flow parameters during operation (Medivators).

**Figure 5:**
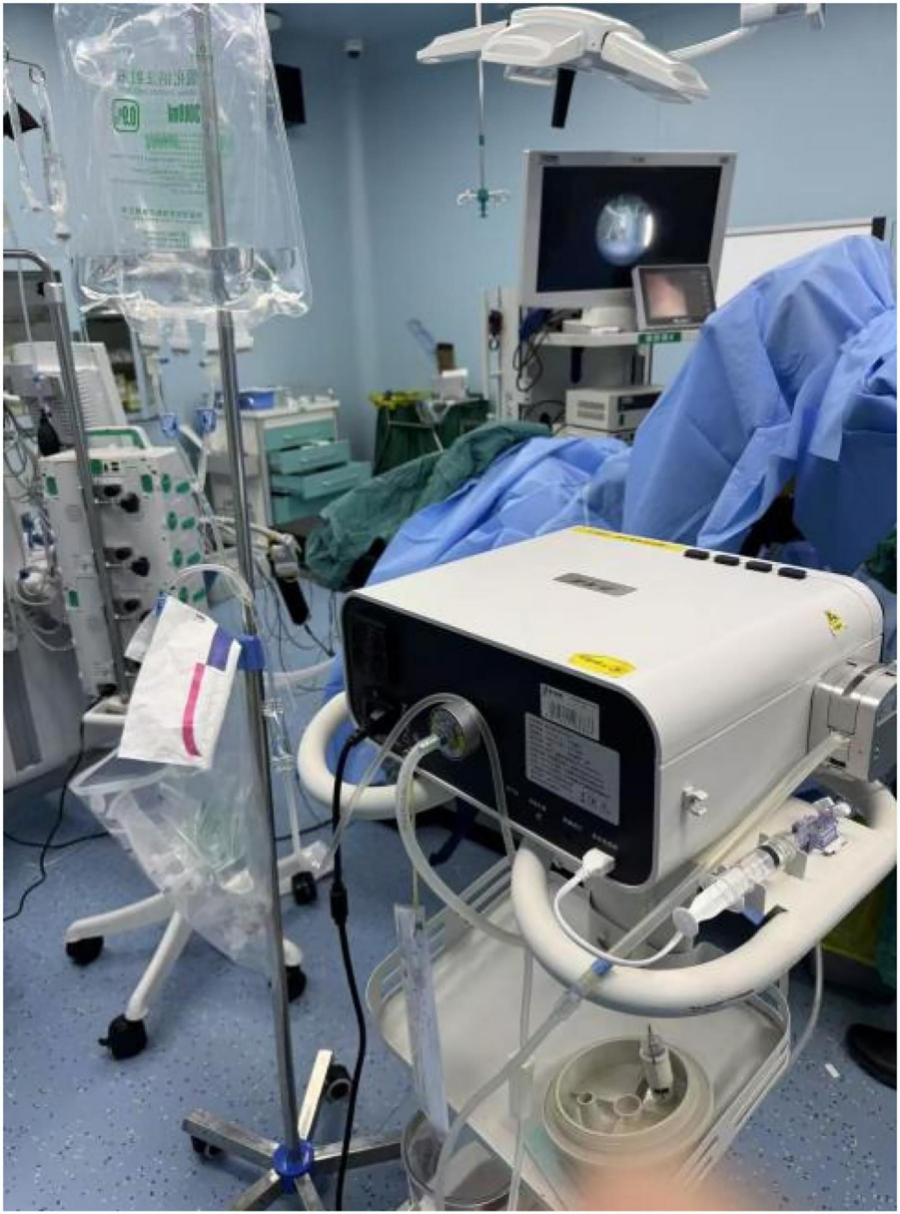
Clinical application of the Medivators infusion-aspiration platform during surgery.

For the experimental group, We carried out intelligent pressure-controlled disposable ureteroscope laser lithotripsy as follows: Under general anesthesia, the patient was positioned in the contralateral decubitus position. This positioning elevates the affected kidney and ureter, allowing gravity to assist in the downward flow of irrigation fluid, which promotes efficient drainage, lowers intrarenal pressure, and improves the operative field clarity. Additionally, it enables the ureter to extend more naturally under gravity, reducing kinking or torsion and facilitating smoother scope advancement. The anatomical alignment also favors more accurate targeting of stones and enhances the removal of fragments. This position may further support optimal respiratory and hemodynamic stability by minimizing thoracic and abdominal compression.1. Using an F8/9.8 WOLF rigid ureteroscope, the pre-placed D-J stent was removed; a COOK malleable guidewire was used for direct renal pelvis entry, followed by the insertion of a 12/14 F Medivators pressure-sensing aspiration ureteral sheath (Jiangxi Yweit Special Technology Co., Ltd. Product Registration Certificate Number: Gan Machinery Approval No. 20192060357), with a length of 320 mm for females and 420 mm for males, was as the working channel. 2. The sheath's infusion, aspiration, and pressure-sensing channels were connected to the Medivators infusion and aspiration platform; We calibrated the pressure-sensing system with water, and the platform was set to automatic mode, with an infusion flow rate of 50–150 ml/min, an intracavitary pressure control value of −15 to −5 mmHg, an alert pressure value of 20 mmHg, and a pressure limit value of 30 mmHg. 3. An F7.5 PUSEN disposable ureteroscope was used to locate the stone. 4. We adjusted Holmium laser settings to 1.0–2.0 J energy and 20–30 Hz frequency; the sheath design and infusion-aspiration platform settings allowed for automatic extraction of powdered fragments through the scope-sheath gap, while larger fragments smaller than the sheath's inner diameter were extracted through negative pressure upon scope withdrawal. 5. We placed a 6F D-J stent before surgery conclusion and left *in situ* for four weeks (Figures 2–5). The pressure-sensing aspiration sheath and its structural components are shown in Supplementary Figures S1 and S2.

### Observation indicators

1. Perioperative Metrics: Evaluation of average operative time, average length of hospital stay, postoperative hemoglobin loss, and renal pelvis pressure between the two groups.
2. Renal Function and Platelets: Assessment of renal function and platelet levels through measurements of blood urea nitrogen (BUN), serum creatinine (Scr), and platelets (PLT).
3. Inflammatory Markers: Evaluation of the inflammatory status *via* white blood cell count (WBC), C-reactive protein (CRP), and procalcitonin (PCT) levels.
4. Stone Clearance: Initial stone clearance rate was defined as the proportion of patients with no detectable residual stones on imaging at the first postoperative assessment. Total stone clearance rate referred to the proportion of patients with complete stone elimination confirmed at the final follow-up. In our protocol, a non-contrast CT scan was performed on the third postoperative day to assess initial clearance, and again one month after discharge and D-J stent removal to evaluate total clearance. Observation of initial stone clearance rate and total stone clearance rate to assess stone removal outcomes in both groups.
5. Complications: We monitored postoperative complications in both groups using the modified Clavien-Dindo classification system. The complications were evaluated using the modified Clavien-Dindo classification system (10), with Grade III and above defined as severe complications. Grade I: No treatment required beyond antiemetics, antipyretics, or analgesics; Grade II: Other pharmacological treatment required, including blood transfusions; Grade III: Surgical, endoscopic or radiological interventions needed, where IIIa does not require general anesthesia and IIIb requires general anesthesia; Grade IV: Life-threatening complications necessitating ICU care; Grade V: Death.

### Statistical methods

Data were analyzed using SPSS software version 25.0. Quantitative data are presented as mean ± standard deviation $\left(\bar{x}\pm s\right)$. Comparisons between groups were conducted using the *t*-test for continuous variables and chi-square test for categorical data. *P*-value < 0.05 was considered statistically significant.

## Results

### Comparison of perioperative metrics between groups

The experimental group had a significantly longer operative time (125.21 ± 10.26 min vs. 110.22 ± 9.19 min, *P* < 0.05), lower renal pelvic pressure (7.61 ± 1.64 vs. 11.07 ± 2.06 cmH_2_O, *P* < 0.05), and shorter hospital stay (5.62 ± 1.26 vs. 6.14 ± 1.48 days, *P* = 0.027).

There was no significant difference in postoperative hemoglobin loss (*P* = 0.06), as shown in Table 2.

**Table 2:** Comparison of perioperative metrics between groups $\left(\bar{x}\pm s\right)$.

Group	*n*	Average operative time (min)	Postoperative hemoglobin loss (g/L)	Renal pelvis pressure (cmH_2_O)	Average hospital stay (days)
Experimental group	70	125.21 ± 10.26	4.11 ± 1.18	7.61 ± 1.64	5.62 ± 1.26
Control group	70	110.22 ± 9.19	4.54 ± 1.39	11.07 ± 2.06	6.14 ± 1.48
*t*		9.10	1.97	10.99	2.238
*P*		<0.05	0.06	<0.05	0.027

“*t*/*χ*^2^” indicates the type of statistical test applied: *t*-values are used for continuous variables (independent samples *t*-test), and *χ*^2^-values are used for categorical variables (Chi-square test). *P*-values <0.05 were considered statistically significant.

### Comparison of renal function and platelets between groups

There was no significant difference in renal function and platelet levels between the two groups pre- and post-operation (*P* > 0.05), as seen in Table 3.

**Table 3:** Comparison of renal function and platelets between groups $\left(\bar{x}\pm s\right)$.

Group	BUN (mmol/L)		Cr (umol/L)		PLT (x10^9^)	
	Pre-operation	Post-operation	Pre-operation	Post-operation	Pre-operation	Post-operation
Experimental group	4.6 ± 1.20	5.14 ± 1.73	87.78 ± 7.36	71.69 ± 4.82	152.32 ± 12.32	142.35 ± 13.16
Control group	5.01.34	6.62 ± 1.52	88.76 ± 7.27	72.24 ± 6.03	154.27 ± 13.51	146.47 ± 12.57
*t*	1.97	1.74	0.79	0.60	0.89	1.89
*P*	0.06	0.08	0.43	0.55	0.37	0.06

“*t*/*χ*^2^” indicates the type of statistical test applied: *t*-values are used for continuous variables (independent samples *t*-test), and *χ*^2^-values are used for categorical variables (Chi-square test). *P*-values < 0.05 were considered statistically significant.

### Comparison of inflammatory markers between groups

Postoperative WBC (5.14 ± 1.03 vs. 6.18 ± 1.86, *P* < 0.05), CRP (28.69 ± 4.82 vs. 48.24 ± 7.03 mg/L, *P* < 0.05), and PCT (0.03 ± 0.01 vs. 0.14 ± 0.03 ng/ml, *P* < 0.05) were all significantly lower in the experimental group, detailed in Table 4.

**Table 4:** Comparison of inflammatory markers between groups $\left(\bar{x}\pm s\right)$.

Group	WBC (×10^12^)		CRP (mg/L)		PCT (ng/ml)	
	Pre-operation	Post-operation	Pre-operation	Post-operation	Pre-operation	Post-operation
Experimental group	5.12 ± 1.20	5.14 ± 1.03	10.78 ± 1.47	28.69 ± 4.82	0.25 ± 0.07	0.03 ± 0.01
Control group	5.43 ± 1.34	6.18 ± 1.86	11.06 ± 1.45	48.24 ± 7.03	0.26 ± 0.06	0.14 ± 0.03
*t*	1.44	6.06	1.13	19.18	0.91	29.10
*P*	0.15	<0.05	0.26	<0.05	0.37	<0.05

“*t*/*χ*^2^” indicates the type of statistical test applied: *t*-values are used for continuous variables (independent samples *t*-test), and *χ*^2^-values are used for categorical variables (Chi-square test). *P*-values <0.05 were considered statistically significant.

### Comparison of stone clearance between groups

Initial stone clearance rate was significantly higher in the experimental group (90.0% vs. 67.1%, *P* < 0.05), as was total clearance rate (95.7% vs. 85.7%, *P* = 0.04). Illustrated in Table 5.

**Table 5:** Comparison of stone clearance outcomes.

Group	n	Initial stone clearance rate	Total stone clearance rate	Number of procedures for stone clearance
Experimental group	70	90.00% (63)	95.71% (67)	1.17 ± 0.48
Control group	70	67.14% (47)	85.71% (60)	2.07 ± 1.06
*t/χ* ^2^		10.87	4.15	6.47
*P*		<0.05	0.04	<0.05

“*t*/*χ*^2^” indicates the type of statistical test applied: *t*-values are used for continuous variables (independent samples *t*-test), and *χ*^2^-values are used for categorical variables (Chi-square test). *P*-values <0.05 were considered statistically significant.

### Comparison of complications between groups

The complication rate was significantly lower in the experimental group (2.86% vs. 21.43%, *P* < 0.05), as shown in Table 6. All complications were classified according to the modified Clavien-Dindo system. In the experimental group, both complications were Grade I. In the control group, 10 events were Grade I (fever, minor infection, ureteral injury), 3 were Grade II (hemorrhage, moderate infection, transfusion), and 2 were Grade I complications due to pleural effusion. No Grade III or higher complications occurred in either group.

**Table 6:** Comparison of complications between groups.

Complication	Experimental Group (*n* = 70)	Control Group (*n* = 70)	*χ* ^2^	*P*
Fever	0	5 (7.14%, 1.43%; Grade I)		
Hemorrhage	0	2 (2.86%; Grade II)		
Sepsis	0	2 (2.86, 1.43%; Grade I)		
Infection	1 (1.43%; Grade I)	2 (2.86%; 1.43%; Grade II)		
Ureteral Injury	1 (1.43%; Grade I)	1 (1.43%; 1.43%; Grade I)		
Transfusion	0	1 (1.43; 1.43%; Grade I)		
Renal Artery Embolism	0	0		
Pleural Effusion	0	2 (2.86%; 1.43%; Grade I)		
Total Complications	2 (2.86%)	15 (21.43%)	13.32	<0.05

“*t*/*χ*^2^” indicates the type of statistical test applied: *t*-values are used for continuous variables (independent samples *t*-test), and *χ*^2^-values are used for categorical variables (Chi-square test). *P*-values <0.05 were considered statistically significant.

## Discussion

Upper urinary tract calculi are common urological conditions (11). Extracorporeal shock wave lithotripsy (ESWL) is widely used due to its non-invasive nature, but it is less effective for stones larger than 2 cm in diameter, staghorn calculi, or stones with high density (12). In such cases, percutaneous nephrolithotomy (PCNL) or ureteroscopy lithotripsy (URSL) may be better options (13, 14). Particularly, URSL, which treats upper urinary tract stones via natural orifices without the need for establishing nephrostomy, is preferred especially for stones smaller than 2 cm in diameter (15). URSL offers significant advantages in treating lower pole renal calculi due to its large range of rotation (16), and thus, it is increasingly used in the treatment of upper urinary tract stones (17). However, during URSL, whether using a perfusion pump or manual syringe irrigation, the high renal pelvic pressure can only be controlled by the surgeon's experience, which may lead to urinary tract infections or perirenal effusion, and even sepsis in severe cases, thereby impacting renal function (18–21). Recent studies, such as that by Mantica et al. have emphasized the risks associated with expanding indications for retrograde intrarenal surgery, highlighting the importance of careful pressure management to prevent complications (22). To address this, a technique named “intelligent pressure-controlled stone clearance system” has emerged. The procedure is performed in the contralateral decubitus position, transitioning from experiential renal pelvic pressure management to intelligent automatic management. Utilizing a pressure-sensing ureteral sheath by Medivators, the system consistently outputs high-flow irrigation fluid and negative pressure suction, allowing flexible control of renal pelvic pressure (23–25). During lithotripsy, stones smaller than 6 mm in diameter can be directly aspirated (23, 24). This approach not only avoids high renal pelvic pressure but also reduces postoperative stone expulsion pain, provides a clearer surgical field, and enhances the safety and stone clearance rate of the surgery. Therefore, this study compares this technology with conventional single-use ureteroscope techniques, aiming to provide clinical reference value for managing upper urinary tract stones.

In this study, the operative time was longer in the experimental group, primarily due to the use of intelligent pressure-controlled suction that enabled repeated irrigation and active retrieval of stone fragments. Unlike the control group, which focused mainly on pulverizing the stones without thorough intraoperative extraction, the experimental approach aimed for more complete stone clearance during the initial surgery. This deliberate and controlled strategy resulted in significantly higher initial and total stone clearance rates, and importantly, did not increase renal burden, as evidenced by stable postoperative BUN and Scr levels. Moreover, the lower incidence of postoperative complications and shorter hospital stay in the experimental group further support the clinical value of this technique despite its longer operative duration. While the intelligent pressure-controlled suction system is designed to work primarily in “dusting” mode rather than with conventional basketing, this did not compromise clinical efficiency in our study. On the contrary, the continuous negative pressure aspiration enabled active removal of stone fragments during lithotripsy, streamlining the procedure and reducing the need for additional maneuvers. Although the operative time in the experimental group was slightly longer, this strategy achieved more thorough stone clearance in a single session and was associated with higher stone-free rates and fewer postoperative complications, indicating that the trade-off between time and outcomes is acceptable in clinical practice.

Although platelet (PLT) levels were recorded as part of routine laboratory monitoring, no significant perioperative changes were observed in either group, and the values remained within normal ranges. This parameter was included to complete the assessment of basic hematologic profiles but did not reflect clinically relevant differences, especially in the absence of sepsis or bleeding complications. The experimental group utilized high-volume irrigation fluid and flexible suction control, reducing renal injury. Notably, We observed significantly lower renal pelvic pressure, postoperative hemoglobin loss, and inflammatory and infection markers (WBC, CRP, PCT), as well as postoperative complications in the experimental group, leading to a shorter average hospital stay. It is worth noting that the average hospital stay in both groups (5.62 ± 1.26 days and 6.14 ± 1.48 days, respectively) appears longer than commonly reported durations in some Western cohorts. This reflects routine institutional practice in our center, where postoperative hospitalization includes scheduled intravenous antibiotic therapy, close monitoring of renal function and infection markers, and imaging (non-contrast CT or KUB) prior to discharge. These measures aim to minimize delayed complications and are influenced by regional medico-legal practices, patient expectations, and the local healthcare system structure. Hence, the surgical approach of the experimental group, which maintains low renal pelvic pressure and perfusion, effectively reduces inflammatory responses and complications, enhancing surgical safety. The high-volume irrigation fluid used in the experimental group was in a circulatory state, maintaining low pressure in the renal pelvis, unlike the relatively closed space of the single-use ureteroscope, which cannot maintain circulation and results in higher renal pelvic pressure. The initial stone clearance rate and total stone clearance rate were significantly higher in the experimental group. These results demonstrate that the experimental group's surgical method has higher therapeutic efficacy, allowing more stones to be cleared in a single operation, thus improving patient quality of life. Our findings are consistent with those reported by Deng et al. (7), who demonstrated that flexible ureteroscopy with pressure-sensing capability significantly reduced renal pelvic pressure and improved stone clearance. Similarly, Chew et al. (21) reported that real-time pressure monitoring during ureteroscopy effectively minimized pressure spikes that could lead to infectious complications. Yang et al. (8) also observed favorable outcomes using a pressure- and temperature-controlled system, with a high stone-free rate and minimal complications. These results support the clinical feasibility of intelligent pressure-controlled systems and reinforce the benefits observed in our experimental group. The effectiveness of the experimental group's surgical method is chiefly attributed to the use of high-volume irrigation fluid and negative pressure suction technology, which can efficiently extract stone fragments, avoiding the “blizzard” effect and ensuring clear surgical visibility. Moreover, the contralateral decubitus position used in the experimental group offers significant advantages over the traditional lithotomy position: it reduces the hydraulic pressure in the ureteral sheath, facilitating stone and flushing fluid expulsion and promoting irrigation fluid circulation. This position also alters the renal axis, placing the renal pelvis in a lower position, decreasing the likelihood of stone displacement due to irrigation. Additionally, in the contralateral decubitus position, the alignment of the urethral internal orifice, the affected ureteral opening, and the renal pelvic outlet nearly forms a straight line, easing sheath insertion. Under this position, effective *in situ* lithotripsy can be achieved, minimizing the risk of stone migration. Even if stones migrate to the middle or upper calyces of the kidney, fine ureteroscopy can follow up for further fragmentation, thereby enhancing the surgery's success rate and safety. Nevertheless, it should be acknowledged that the positioning strategy may have contributed independently to some of these favorable outcomes. Although the intelligent pressure-controlled system was the main focus of evaluation, the contralateral decubitus position could have synergistically lowered renal pelvic pressure and improved irrigation dynamics. This implies that part of the observed benefit may reflect a combined effect of positioning and pressure-control technology, underscoring the need for future randomized studies with standardized positioning to better isolate the specific role of each factor.

From an economic perspective, although the intelligent pressure-controlled disposable ureteroscope system involves higher initial procedural costs due to specialized equipment, its potential long-term cost-effectiveness should be interpreted cautiously. The reduction in complication rates, shorter hospitalization, and higher stone clearance efficiency observed in this study may translate into downstream savings, yet such implications remain inferential and require confirmation through dedicated cost-effectiveness analyses. Accordingly, while this technique may hold promise for improving both clinical outcomes and healthcare resource utilization, further health-economic studies are warranted before definitive conclusions can be drawn. However, as this was a retrospective and non-randomized study, the observed advantages should be interpreted as associations rather than definitive evidence of superiority.

## Limitations

This study has several limitations. First, it was conducted at a single center, which may limit the generalizability of the results to other clinical settings. Second, although the sample size was sufficient for detecting significant differences in common surgical outcomes, it may be inadequate to assess less frequent adverse events. Third, the non-randomized design may introduce selection bias, even though baseline characteristics were balanced between the two groups. In addition, detailed anatomical locations of the stones (e.g., renal pelvis, upper/middle/lower calyx) were not uniformly recorded across all patients, which prevented further subgroup analysis based on stone position. Future studies should not only focus on validating the effectiveness of this technology for upper urinary tract stones but also explore its applications in treating chronic ureteral strictures and complex renal stones. Additionally, comparisons with PCNL and RIRS could provide valuable insights into the most effective treatment strategies for various stone types and locations. Future multi-center, randomized controlled studies with larger cohorts are necessary to further validate the clinical efficacy and safety of intelligent pressure-controlled disposable ureteroscopes. Moreover, due to the retrospective nature of this study, causality cannot be established, and residual confounding may remain despite matching.

## Conclusions

In conclusion, in this retrospective matched cohort study, the use of the contralateral decubitus position combined with intelligent pressure-controlled ureteroscopic stone extraction was associated with reduced postoperative inflammatory markers, lower renal pelvic pressure, and improved stone clearance rates compared to conventional techniques.

This technique also showed a lower rate of postoperative complications and shorter hospital stays. While these findings suggest potential clinical advantages, they should be interpreted with caution due to the retrospective, non-randomized, single-center nature of the study.

Further prospective, multicenter randomized controlled trials are warranted to validate these results and determine the broader applicability of this approach.

## Data Availability

The datasets presented in this study can be found in online repositories. The names of the repository/repositories and accession number(s) can be found in the article/Supplementary Material.

## Supplementary material

Supplementary Figure S1 Appearance and structural components of the intelligent pressure-controlled ureteric sheath. (A) Intelligent Pressure-Controlled Sheath + Inner Core (B) Magnified View of the Intelligent Pressure-Controlled Sheath (C) Disassembled View of the Intelligent

Supplementary Figure S2 Components of the intelligent pressure monitoring system. (A) Pressure Measurement Tube (B) Pressure-Sensing Transducer
