# Supplementary figures and images for: Clinical outcomes of an intelligent pressure-controlled disposable ureteroscope for Laser lithotripsy in renal stone surgery: a retrospective matched cohort study
Source: Front Surg. 2025 Oct 28;12:1637385. doi: 10.3389/fsurg.2025.1637385 (PMC12602390; doi:10.3389/fsurg.2025.1637385)

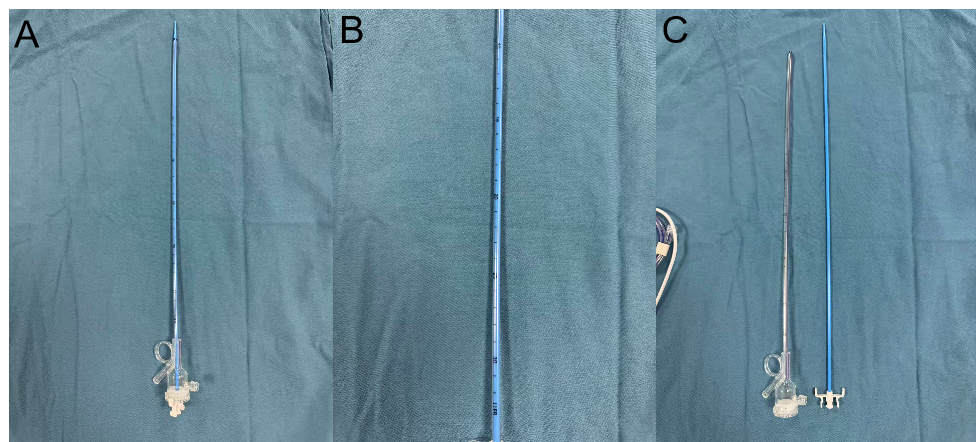

Supplement: Supplementary Figure S1 — Appearance and structural components of the intelligent pressure-controlled ureteric sheath. (A) Intelligent Pressure-Controlled Sheath + Inner Core (B) Magnified View of the Intelligent Pressure-Controlled Sheath (C) Disassembled View of the Intelligent [file Image1.tif]

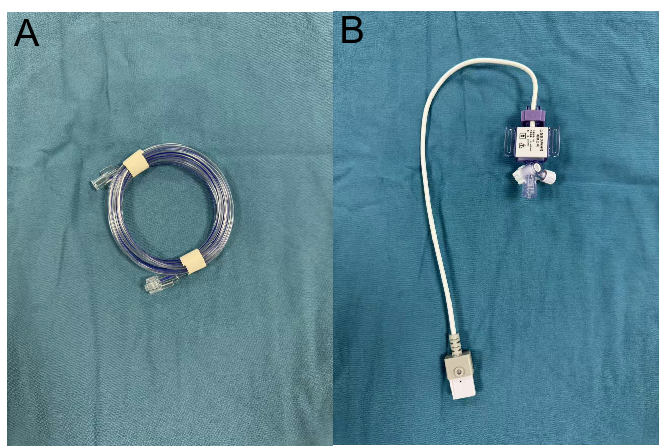

Supplement: Supplementary Figure S2 — Components of the intelligent pressure monitoring system. (A) Pressure Measurement Tube (B) Pressure-Sensing Transducer [file Image2.tif]
